# Sudden massive neck swelling due to hemorrhage of a thyroid adenoma: a case report

**DOI:** 10.1186/1752-1947-5-391

**Published:** 2011-08-18

**Authors:** Evangelos I Giotakis, Tanja Hildenbrand, Joachim Dodenhöft

**Affiliations:** 1 ENT Department, Städtisches Klinikum Karlsruhe, Moltkestrasse 90, 76133, Karlsruhe, Germany

## Abstract

**Introduction:**

Sudden swelling of the neck is an emergency situation that can be life-threatening for the patient. Therefore, an understanding of the possible underlying pathology is of great importance. Sudden massive swelling of the neck because of intralesional bleeding of a thyroid adenoma is seldom encountered but must be considered. Such massive swelling caused by spontaneous bleeding of a thyroid adenoma has not yet been described in the literature.

**Case presentation:**

We report the case of a 71-year-old Caucasian man with sudden massive neck swelling due to intralesional bleeding of a thyroid adenoma. We present his clinical history, physical examination results, computed tomography (CT) scans, and histological findings after surgery. He presented with sudden massive swelling of the left side of his neck after sneezing while working with his hands over his head. An ear, nose, and throat examination showed a painless swelling of the left side of his neck and a displacement of his larynx to the right. CT scans revealed a mass originating from the left lobe of his thyroid gland and the mass displaced his larynx and trachea. A surgical exploration showed a greatly enlarged left lobe of his thyroid gland. A histopathological examination showed a hemorrhagic infarction of a follicular thyroid adenoma.

**Conclusions:**

Sudden intralesional bleeding of a thyroid adenoma is a rare condition but one that should be considered in cases of sudden and massive swelling of the neck.

## Introduction

Follicular adenomas, arising from the thyroid follicles, are the most common benign tumors of the thyroid gland. They are more common in women than in men and occur in all age groups. Most adenomas are inactive and appear as cold nodules on scintigraphy images. However, autonomous adenomas, which can produce thyroid hormone without the regulation of thyroid-stimulating hormone, appear as hot nodules on scintigraphy images.

As long as the patient is euthyroid, the clinical symptoms are limited to a palpable neck swelling that depends on the size of the adenoma. If hyperthyroidism develops, other typical clinical symptoms such as weight loss, hair loss, restlessness, palpitation, sweating, thirst, and diarrhea can be observed. If the blood supply to an adenoma is insufficient, the tumor can stop growing and involute. Adenomas can also become necrotic and calcified.

## Case presentation

A 71-year-old Caucasian man was admitted with sudden swelling of the left side of his neck to the emergency department of our ear, nose, and throat clinic. The swelling occurred after he sneezed while lying on his back and working with his hands over his head. He reported that he had noticed a slight painless swelling of the neck a few months before. He did not complain of dyspnea or pain.

**Figure 1:**
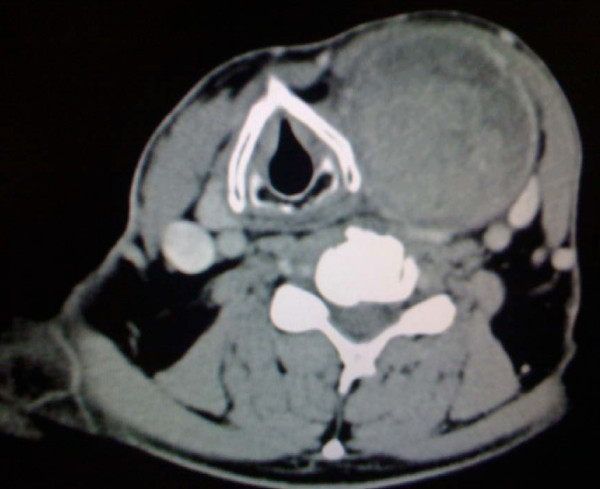
**Axial computed tomography (CT) image showing the displacement of the larynx to the right and the inhomogeneous mass on the left**.

**Figure 2:**
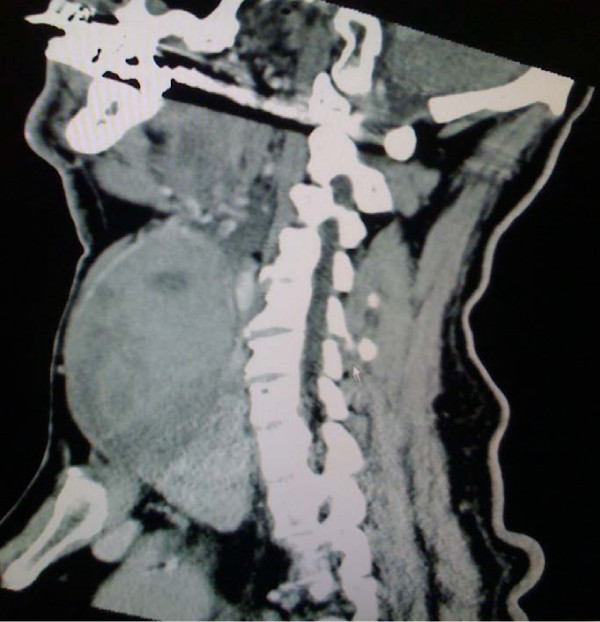
**Sagittal computed tomography (CT) image showing the extent of the mass and the contrast enhancement in its caudal region**.

**Figure 3:**
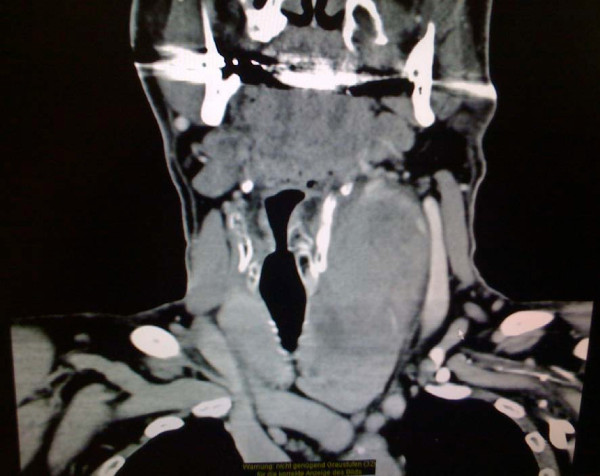
**Coronal computed tomography (CT) image showing the possible connection of the mass to the thyroid gland**.

A clinical examination showed a painless mass in the left side of his neck but no signs of inflammation or ecchymosis. The semi-hard swelling extended from his clavicle to the submandibular space. Indirect laryngoscopy with a 70° scope showed a displacement of his larynx to the right and normal vocal cord function. Blood tests revealed no signs of inflammation, and his white blood cell count was 4900 cells/μL. His hemoglobin and blood calcium levels were normal (14.1 g/dL and 9.6 mg/dL, respectively). An ultrasound of his neck showed a heterogeneous, well-defined mass that was located medial to his sternocleidomastoid muscle and that displaced his carotid artery and jugular vein. Computed tomography (CT) scans of his neck (Figure 1, Figure 2 and 3) showed a large mass that measured approximately 6 × 7 × 12 cm and originated in the left lobe of his thyroid gland. The mass displaced his larynx and trachea to the right and extended from the subclavicular to the submandibular region. There was contrast enhancement in the capsule and caudal part of the mass.

**Figure 4:**
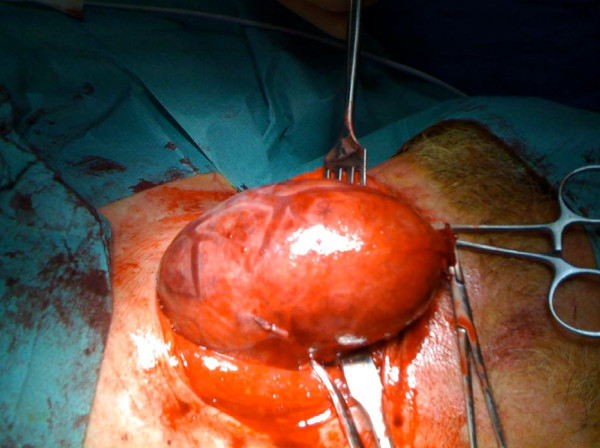
**Intraoperative dissection showing the intact mass and the sternocleidomastoid muscle to the side**.

**Figure 5:**
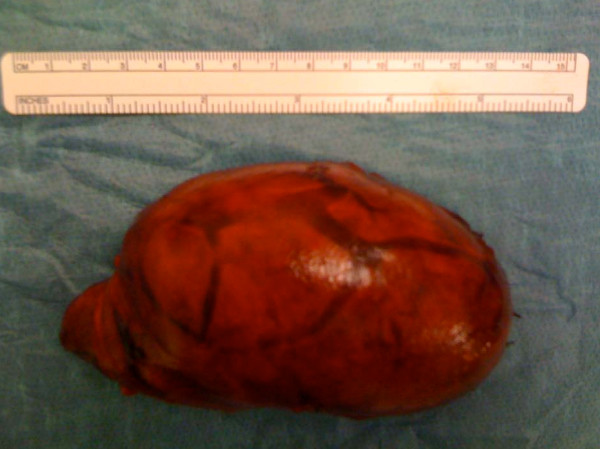
**Left lobe of the thyroid gland after dissection**.

A surgical exploration of his neck revealed a mass that was located medial to his sternocleidomastoid muscle and anterior to his jugular vein (Figure 4). The mass was easily dissected from the surrounding structures and was found to originate in his thyroid gland. A hemithyroidectomy was performed (Figure 5). His laryngeal nerve was recognized and preserved. A histopathological examination revealed a follicular thyroid adenoma with a hemorrhagic infarction.

## Discussion

Sudden lateral swelling of the neck is typically caused by an acute inflammatory reaction. Spontaneous bleeding in the thyroid gland is not a common clinical occurrence but can be life-threatening, and the diagnosis can be challenging. The differential diagnosis should include the following pathologies: laryngocele, lateral neck cyst infection, neck abscess, retropharyngeal abscess, soft tissue edema, aneurysm, submandibular gland swelling, and thyroid or parathyroid gland [1-7]. In this case, because our patient noticed the swelling after sneezing, a laryngocele was considered first in the differential diagnosis. This diagnosis was excluded on the basis of the CT scan because the huge mass had no connection to his larynx and no air was found in the neck spaces. If the imaging results reveal a solid mass, fine-needle aspiration should precede exploration of the neck to exclude malignancy first. An infectious cause was also excluded because there were no clinical signs of inflammation (no pain upon palpation and no skin redness or edema). An aneurysm was also excluded from the differential diagnosis on the basis of the CT scan and the ultrasound of the neck. The differential diagnosis included the spontaneous rupture of a parathyroid adenoma, accompanied by an extracapsular hemorrhage [8,9]. In such cases, severe abnormalities in the blood calcium levels are typically observed. Sudden lateral neck swelling may also be caused by fine-needle aspiration [10,11]. Another rare but possible cause of sudden cervical swelling is a secondary hemorrhage of a parathyroid carcinoma [5]. This pathology coexists with abnormal calcium levels. A case of lateral neck swelling caused by anaplastic thyroid cancer has also been described in the literature [8]. Rapidly progressive malignancies, such as lymphomas or sarcomas, could manifest with similar clinical characteristics.

After collecting the data described above, we decided to proceed with exploratory surgery on the day after our patient was hospitalized. Emergency surgery on the day of admission was not indicated, because he showed no signs of dyspnea or stridor. In a case of displacement of the larynx and trachea, the surgeon should also be aware of the possible risk of intubation failure and inform the anesthesiologist of the laryngeal status of the patient. Additionally, a fiberoptic intubation device and an emergency tracheostomy set should always be available. The surgery was performed with minimal bleeding and a short surgical time (approximately 45 minutes).

## Conclusions

With a precise clinical history and proper radiological examinations, exploratory surgery to identify sudden intralesional bleeding of the thyroid gland can be a safe and rapid method of treatment and minimize complications.

## Abbreviations

CT: computed tomography.

## Consent

Written informed consent was obtained from the patient for publication of this case report and any accompanying images. A copy of the written consent is available for review by the Editor-in-Chief of this journal.

## Competing interests

The authors declare that they have no competing interests.

## Authors' contributions

EIG analyzed and interpreted the patient data and performed the surgery. TH analyzed and interpreted the patient data. JD performed the surgery. All authors read and approved the final manuscript.

## References

[B1] Tana M Leeb F Clarkec M Thoracic aneurysm rupture presenting as a rapidly enlarging neck mass Eur J Emerg Med 2006 13 182 183 10.1097/01.mej.0000188226.22239.59 16679887

[B2] Garrett HE Heidepriem RW Broadbent LP Ruptured aneurysm of the inferior thyroid artery: repair with coil embolization J Vasc Surg 2005 42 1226 1229 10.1016/j.jvs.2005.08.004 16376220

[B3] Taniguchi I Maeda T Morimoto K Miyasaka S Suda T Spontaneous retropharyngeal hematoma of a parathyroid cyst: report of a case Surg Today 2003 33 354 357 10.1007/s005950300080 12734730

[B4] Merante-Boschin I Fassan M Pelizzo MR Casal E Rugge M Neck emergency due to parathyroid adenoma bleeding: a case report J Med Case Reports 2009 3 7404 10.1186/1752-1947-3-7404 19830200 PMC2726549

[B5] Erdas E Licheri S Lai ML Pisano G Pomata M Daniel GM Cervico-mediastinal hematoma secondary to extracapsular hemorrhage of parathyroid carcinoma. Clinical case and review of the literature Chir Ital 2003 55 425 434 12872580

[B6] Herman D Piller P Kennel P Stierle JL Conraux C Extensive cervical hematoma complicating multinodular goiter. Apropos of a case Ann Otolaryngol Chir Cervicofac 1992 109 105 107 1524359

[B7] Chang CC Chou YH Tiu CM Chiou HJ Wang HK Chiou SY Chen SP Chang MW Chang CH Hong TH Spontaneous rupture with pseudoaneurysm formation in a nodular goiter presenting as a large neck mass J Clin Ultrasound 2007 35 518 520 10.1002/jcu.20314 17486567

[B8] Haas V Celakocsky P Brtkova J Hornychova H Unusual manifestation of anaplastic thyroid cancer Acta Medica 2008 51 233 236 19453090 10.14712/18059694.2017.30

[B9] Tagliaferro P Talamo C Chini L Mangiola A Massive intra-thyroid hemorrhage in a patient with adenoma Minerva Med 1980 71 1693 1700 7383425

[B10] Roh JL Intrathyroid hemorrhage and acute upper airway obstruction after fine needle aspiration of the thyroid gland Laryngoscope 2006 116 154 156 10.1097/01.mlg.0000187396.18016.d0 16481831

[B11] Park MH Yoon JH Anterior neck hematoma causing airway compression following fine needle aspiration cytology of the thyroid nodule: a case report Acta Cytol 2009 53 86 88 10.1159/000325089 19248559

